# Supplementary material for: Momentum-dependent power law measured in an interacting quantum wire beyond the Luttinger limit
Source: Nat Commun. 2019 Jun 27;10:2821. doi: 10.1038/s41467-019-10613-2 (PMC6597579; doi:10.1038/s41467-019-10613-2)
Supplement: Supplementary file 1 — Supplementary Information [file 41467_2019_10613_MOESM1_ESM.pdf]

SUPPLEMENTARY INFORMATION

Momentum-dependent power law measured in an interacting quantum wire beyond the Luttinger limit

Jin *et al.*

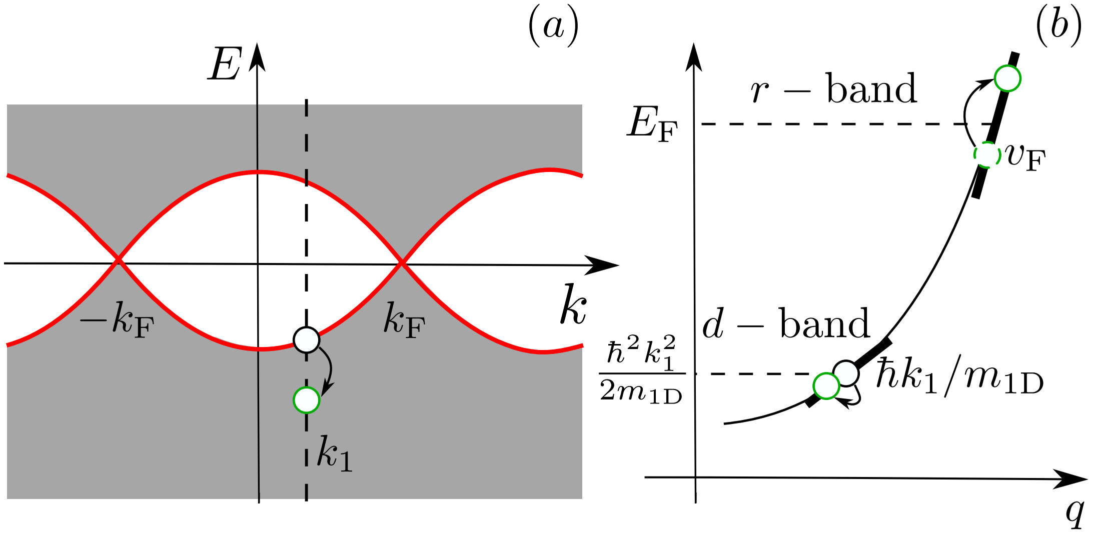

Supplementary Figure 1 | Excitations of an interacting 1D system. (a) Dispersion of an interacting 1D system. White is the kinematically forbidden region (see explanation in the text) and grey is the continuum of many-body excitations. The thick red line separates the border between the two regions. The states on the border that form it correspond to removing a single particle (marked by a black circle at k_1) from the many-particle state, and an excitation described by the mobile-impurity model (marked by a green circle). (b) Splitting of the fermionic dispersion into two subbands, one for the heavy hole with velocity $\hbar k_1/m_{1D}$ and one for the excitations around E_F with velocity v_F. The green circles are the constituent parts of the many-body excitations in the nonlinear regime (see explanation in the text).

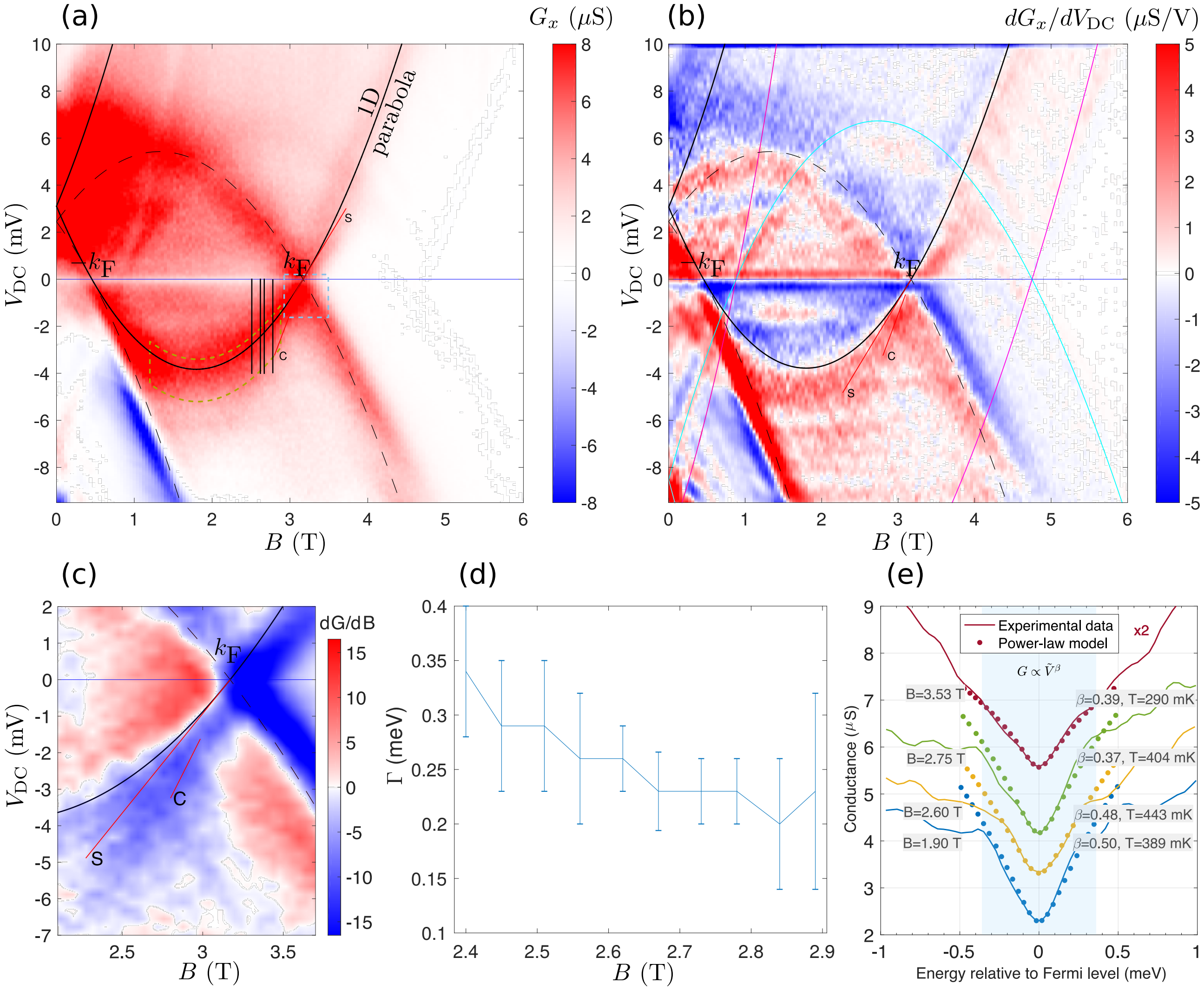

Supplementary Figure 2 | Detailed plots of data for sample B, matching those shown in the paper for sample A.
(a) Density plot of G (with correction for the parasitic conductance). (b) $\mathrm{d}G/\mathrm{d}V_{\mathrm{DC}}$ without any correction. (c) Zoom-in on $\mathrm{d}G/\mathrm{d}B$ in the spin-charge-separation region. (d) Γ_{I} *vs* B. (e) Power-law fits for the ZBA. The sample temperature was about 330 mK.

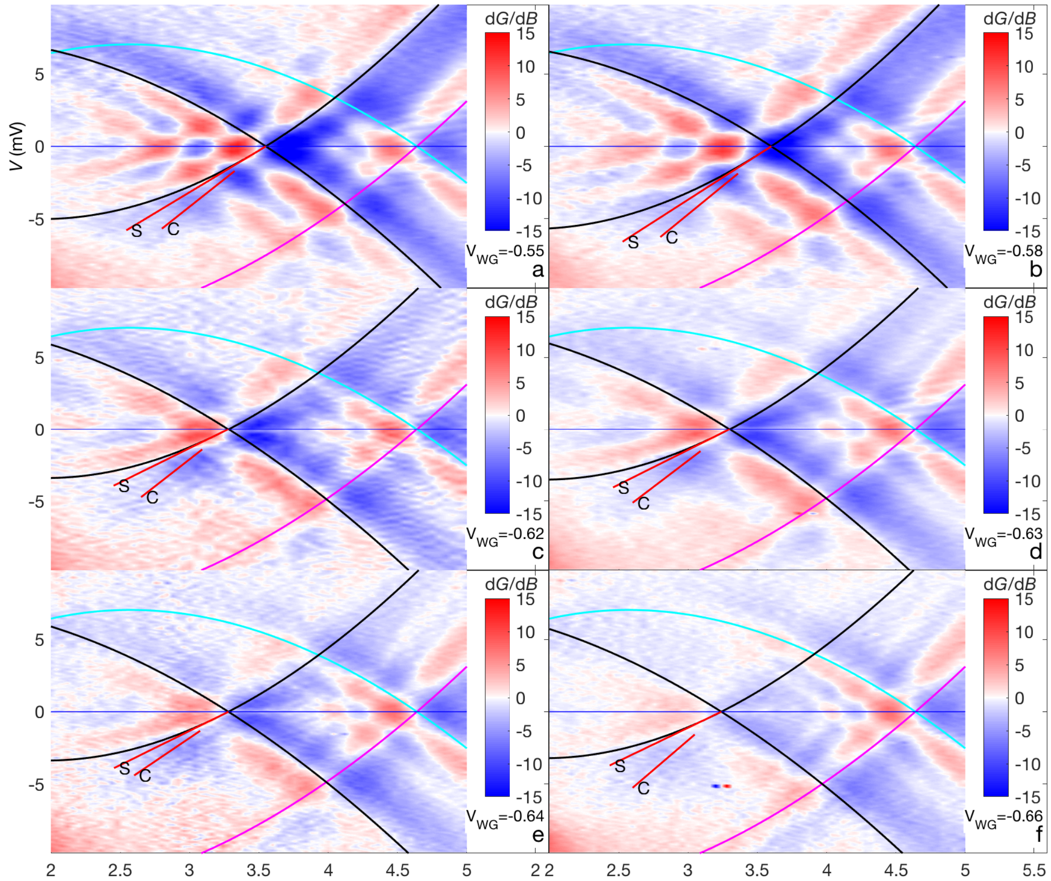

Supplementary Figure 3 | Plots of differential conductance with respect to B at various gate voltages. All measurements were conducted at 150 mK on sample A. The spin and charge lines are marked S and C, respectively, and used to extract the corresponding values of K_{c} given in Supplementary Table I. The parabolae were used to calculate the electron densities of the upper (n_1) and lower (n_2) quantum wells, which are also shown in the table. The corresponding results for sample B are given in Fig. 8 and Table II of Ref. [1].

Supplementary Table I | Charge- and spin-wave velocities extracted from figure 3 for sample A.

**Subfigure label**	$\mathbf{V}_{\mathrm{WG}}$ /V	$\mathbf{n_1}$ $/10^7\,\mathrm{m}^{-1}$	$\mathbf{n_2}$ $/10^{15}\,\mathrm{m}^{-2}$	$\mathbf{v}_{\mathrm{s}}$ $/10^5\,\mathrm{m\,s}^{-1}$	$\mathbf{v}_{\mathrm{c}}$ $/10^5\,\mathrm{m\,s}^{-1}$	$\mathbf{K_c} = \mathbf{v}_{\mathrm{s}}/\mathbf{v}_{\mathrm{c}}$
a	-0.55	5.16	1.85	1.65	2.17	0.76
b	-0.58	5.50	1.76	1.76	2.20	0.80
c	-0.62	4.25	1.85	1.36	2.15	0.63
d	-0.63	4.32	1.85	1.38	2.09	0.66
e	-0.64	4.23	1.85	1.35	1.85	0.73
f	-0.66	4.13	1.84	1.32	2.35	0.56

Supplementary Table II | Charge- and spin-wave velocities extracted from data reported in previous work (Fig. 8 in [1]) for sample B.

**Subfigure label**	$\mathbf{V}_{\mathrm{WG}}$ /V	$\mathbf{n_1}$ $/10^7\,\mathrm{m}^{-1}$	$\mathbf{n_2}$ $/10^{15}\,\mathrm{m}^{-2}$	$\mathbf{v}_{\mathrm{s}}$ $/10^5\,\mathrm{m\,s}^{-1}$	$\mathbf{v}_{\mathrm{c}}$ $/10^5\,\mathrm{m\,s}^{-1}$	$\mathbf{K_c} = \mathbf{v}_{\mathrm{s}}/\mathbf{v}_{\mathrm{c}}$
a	-0.60	5.68	1.67	1.54	2.29	0.67
b	-0.65	4.99	1.65	1.36	2.14	0.64
c	-0.68	4.79	1.52	1.30	1.91	0.68
d,e	-0.70	4.60	1.48	1.39	2.28	0.61

Supplementary Note 1. Other possible causes of conductance enhancement

It is important to exclude other possible causes of the enhancement of tunnelling conductance below the 1D subband edge. We have already removed a background parasitic conductance. In Fig. 4, the dashed lines show the conductance where this background has been increased by 20% (allowing for a slight reduction in area of the parasitic region with gate voltage) or decreased by 20% (allowing for a remnant of the 1D signal in the background). This variation reflects the uncertainty in the background-elimination process, arising from the slight change in density and area of the p region when the wire gates are pinched off. The enhancement above the conductance predicted by models 1 and 3 is still significant, whereas there is still a good fit to model 2. As the 1D wires are squeezed, minute lithographic imperfections and occasional impurities will cause fluctuations in the wire width and potential depth. When the wires are nearly pinched off, tunnel barriers may form across a wire, producing localised quantum-dot states. Given that the tunnelling probability across the 14 nm $Al_{0.33}Ga_{0.67}As$ barrier between the array of 1D wires and the 2D layer is very low, the wires are likely to remain equipotentials despite possibly being broken up into segments in some places.

The effect of this small disorder on electrons is strongly amplified in 1D according to the one-parameter scaling theory[2], resulting in a fully (Anderson) localised [3] single-particle spectrum in the thermodynamic limit. For a system of finite length, however, above some energy (mobility) threshold the energy-dependent localisation length exceeds the sample length [4, 5], e.g. as was observed directly in cold-atom experiments [6]. In this experiment (see Fig. 5(a)), Γ_{I} increases sharply for $B < 2.5\,\mathrm{T}$, corresponding to band energies below the mobility threshold ($\simeq 0.18 E_{\mathrm{F}}$ above the bottom of the band here), but above this field we have access to the nonlinear TLL physics in the rest of the 1D band. Note that in the interacting model, the states below the 1D parabola are not single-particle states but many-body states (Fig. 2(d)). The disorder below the mobility threshold therefore does not affect the many-body states directly since they are formed by higher-momentum constituents.

Note that there are some horizontal streaks in Fig. 2(a) and 2(b), which contribute only about 5% of the overall conductance in most measurements and can be seen as small fluctuations on the curves in Fig. 4. They must be caused by tunnelling via localised states where k_x is no longer a good quantum number. As they are seen in a wide range of energies uncorrelated with the 1D band, they may arise from occasional defects or large clusters of donors where the depth of the potential may be as low as 10 meV below the Fermi level. If such states were the cause of the observed excess in signal which we attribute to interactions (Fig. 4), the fact that this is strongly B-dependent would require a sharp cutoff in the size distribution of these localised states or dots. The excess changes rapidly between 2.4 and 2.6 T, and if we estimate this to be when the length of the dot becomes comparable to the magnetic length $l_{\mathrm{B}} = \sqrt{\hbar/eB}$, with $l_{\mathrm{B}} = 16.5\,\mathrm{nm}$ at 2.4 T and 15.9 nm at 2.6 T, there must be dots of sizes down to 16.0 nm but far

fewer just below this. Hence there is a particular size distribution, which is very unlikely. The small contribution from the observed horizontal streaks is just superimposed on top of the 1D signal and does not affect the model.

At low energy the universal behaviour of the linear TTL emerges in the limit of vanishing momentum around the Fermi point. At any finite momenta away from it the correlation functions of the TTL model can be modified by the momentum dependence of a finite-range two-body interaction potential[7, 8] or by m^{-1} corrections of the originally parabolic single-particle dispersion[9, 10], altering the constant power-law behaviour of conductance. In order to assess their contribution we fit a constant power law predicted by the standard TTL at low energy and find no observable deviations from it in a finite range of energies around $E_{\rm F}$, (the blue shaded regions in Figs. 4 and 5(b)). Thus, any corrections to the standard TTL model in this range of energies are negligible.

Supplementary Note 2. Modelling the 1D spectral function in nonlinear regime

In 1D, the theoretical description of the many-body excitations away from the Fermi points is given by the nonlinear TTL theory [11], which we call model 2. It is instructive to consider the original model of interacting fermions first. Any two-body interaction changes the delta-function excitation spectrum of the free particles (centred at the single-particle parabola) into a continuum, since removal of a single particle from the system affects all other particles, by involving their many degrees of freedom. In two and three dimensions this continuum covers the whole energy-momentum space since it is always possible to create an excitation at an infinitesimally small energy at all finite momenta by connecting two points on a circle or on a sphere by a finite vector of length $|\mathbf{k}| < k_F$. The fact that there are only two Fermi points in 1D makes it very special. There is a minimal energy for removing a single particle, with a finite k. The process corresponds to taking out just this one particle without touching the rest (shown as a black circle in Supplementary Figure 1(a)). This leads to a forbidden region on the energy-momentum plane (see white regions in Supplementary Figure 1(a)), which are separated from the many-body continuum of the excitations by a line that is given by the dispersion of single hole with the minimal energy (see thick red line in Supplementary Figure 1(a)).

Away from the Fermi energy, excitations around this line are described by nonlinear hydrodynamics. A hole state deep under the Fermi surface is reminiscent of another problem—X-ray scattering in metals, where the deep hole is created by absorption of a high-energy X-ray photon. This system is known to have power-law singularities close to the Fermi level that originates from the interactions between the deep hole and the quasiparticles around $E_{\rm F}$. Although the standard perturbation theory in the interaction for the X-ray problem is divergent, a way of handling these divergences was proposed by Nozières and De Dominicis [12] in the form of the heavy-impurity model. This model, consisting of the Fermi-liquid quasiparticles interacting with a localised state deep under the Fermi level, can be diagonalised exactly, accounting for all divergences in all orders of perturbation theory. It predicts power-law behaviour around $E_{\rm F}$. An analogous construction of a mobile-impurity model can be done in 1D starting from the Tomonaga-Luttinger model [13],

$$H_{\rm TLL} = \frac{\hbar v_c}{2\pi}\int {\rm d}x \left[K_c \left(\nabla\theta_c\right)^2 + \frac{1}{K_c}\left(\nabla\varphi_c\right)^2\right] + \frac{\hbar v_s}{2\pi}\int {\rm d}x \left[K_s \left(\nabla\theta_s\right)^2 + \frac{1}{K_s}\left(\nabla\varphi_s\right)^2\right], \qquad (1)$$

where θ_α and φ_α are canonically conjugated variables $[\varphi_\alpha(x), \nabla\theta_\beta(x')] = i\pi\delta_{\alpha\beta}\delta(x-x')$ that describe the charge-density wave (CDW) $\alpha = c$ and the spin-density wave (SDW) $\alpha = s$, and v_α and K_α are the Luttinger parameters of these modes that are input parameters of the model. Their values have to be specified for a particular system. Below we consider a strictly one-dimensional system omitting the spatial direction index that we use in the main text, $k = k_x$, for brevity and keep K_α, v_α arbitrary for generality.

A well-defined deep-hole state can be added to $H_{\rm TTL}$ assuming that the hole state is sufficiently far in energy from the Fermi level (see the construction in Supplementary Figures 1(a) and (b)) that it is not dynamically created or annihilated by the low-energy excitations. The dispersion of this hole is now an arbitrary input parameter of the model, $\varepsilon(k)$, describing phenomenologically the dispersion of the spectral edge, see the red line in Supplementary Figure 1(a). Its coupling to the CDW of the linear Luttinger liquid is of the density-density type since exchange processes are forbidden by a larger energy difference between the hole band and $E_{\rm F}$, see Supplementary Figure 1(b). The mobile impurity does not couple to the spin modes in an unpolarised TLL owing to symmetry of the two spin orientations. The linear Tomonaga-Luttinger model, a mobile impurity, and coupling between them together form

the nonlinear TLL model (also called the mobile-impurity model) [14, 15],

$$H_{\mathrm{nTLL}} = H_{\mathrm{TTL}} + \int dx\, d^{\dagger}(x) \left(\varepsilon(k) - i\frac{\partial \varepsilon(k)}{\partial k}\nabla\right) d(x) + \int dx \left(V_{\theta}\nabla\theta_c + V_{\varphi}\nabla\varphi_c\right) d^{\dagger}(x)\, d(x)\,, \tag{2}$$

where the fermionic operator $d(x)$, satisfying $\left\{d(x), d^{\dagger}(x')\right\} = \delta(x - x')$, models a deep hole state, and the coupling constants V_θ and V_φ are also not independent parameters. They can be related to the linear Luttinger parameter and the dispersion of the spectral edge by considering the velocity of the whole system and the variation of the total energy with the density, which fixes the couplings as [16]

$$V_{\theta} = \frac{\hbar^2 k}{\sqrt{2}m_{\mathrm{1D}}} - \frac{\partial\varepsilon(k)}{\sqrt{2}\partial k}, \tag{3}$$

$$V_{\varphi} = \frac{\hbar\partial\varepsilon(k)}{\sqrt{2}\partial\rho} + \frac{\hbar v_s}{\sqrt{2}K_s}. \tag{4}$$

The validity of the nonlinear TTL model is restricted to the proximity of the spectral edges [17]. Moving a deep hole down in energy (for example in the hole sector) away from the the spectral threshold (see the green circle in Supplementary Figure 1(a)) requires creation of CDWs with higher and higher energy (see green circles in the r-band in Supplementary Figure 1(b)) according to the model in Supplementary Eq. (2). However, for sufficiently large energies, the original linear approximation of the Tomonaga-Luttinger model becomes invalid, which also voids the validity of the nonlinear model. Due this constraint the dispersion of mobile impurity in the second term in Supplementary Eq. (2) is also linearised around a given momentum k defining a linear impurity subband (see d-band in Supplementary Figure 1(b)) and simplifying diagonalisation of the nonlinear model. The nonlinear excitations around the spectral edge, described by the mobile-impurity model in Supplementary Eq. (2), are composite many-body states consisting of a deep hole and a relatively small number of CDWs around the around E_{F} (see all green circles in Supplementary Figure 1(b)). The nonlinear TTL model also becomes undefined close to the Fermi energy, where CDWs can create many holes at the spectral edge and strongly hybridise with them dynamically, qualitatively altering the structure of the model in Supplementary Eq. (2).

The mobile-impurity model in Supplementary Eq. (2) can be diagonalised using a unitary rotation in the two-by-two space of the Tomonaga-Luttinger model and the mobile impurity. Then, the expectation values for the observables can be evaluated using the Gaussian integrals over the free fields, as for the linear Tomonaga-Luttinger liquid. The diagonalisation is performed via the $e^{-iU}H_{\mathrm{nTLL}}e^{iU}$ rotation, where the rotation matrix can be found in the perturbation-theory analysis from the condition $[H, U] = 0$ as

$$U = \int \mathrm{d}x \left[C_{+}\left(\sqrt{K_{\mathrm{c}}}\theta_{\mathrm{c}} + \varphi_{\mathrm{c}}/\sqrt{K_{\mathrm{c}}}\right) + C_{-}\left(\sqrt{K_{\mathrm{c}}}\theta_{\mathrm{c}} - \varphi_{\mathrm{c}}/\sqrt{K_{\mathrm{c}}}\right)\right] d^{\dagger}(x)\, d(x)\,, \tag{5}$$

where the coefficients are

$$C_{\pm} = \frac{\frac{\hbar(k-k_{\mathrm{F}})}{m_{\mathrm{1D}}\sqrt{K_c}} \pm \sqrt{K_{\mathrm{c}}}\left(\frac{2}{\pi}\frac{\partial\varepsilon(k)}{\partial\rho} + \frac{\partial\varepsilon(k)}{\hbar\partial k}\right)}{2\sqrt{2}\left(\frac{\partial\varepsilon(k)}{\hbar\partial k} \mp \frac{\hbar k_{\mathrm{F}}}{m_{\mathrm{1D}}K_{\mathrm{c}}}\right)}, \tag{6}$$

and the coupling constant from Supplementary Eqs. (3,4) have already been substituted.

The averages with respect to the free model after the rotation can be evaluated as integrals over the free bosonic and fermionic fields. We will consider only the spectral function here. It can be defined using the Green function as [18]

$$A_1(k, E) = -\frac{1}{\pi}\mathrm{Im}G_{\alpha\alpha}(k, E)\,\mathrm{sign}E, \tag{7}$$

where the real-frequency Green function is a Fourier transform of the time-ordered two-point correlation function, $G_{\alpha\beta}(k, E) = -i\int \mathrm{d}x\mathrm{d}t \exp(iEt/\hbar - ikx)\left\langle T\psi_{\alpha}(x,t)\,\psi_{\beta}(0,0)\right\rangle$. Since the model H_{nTLL} is $SU(2)$ symmetric in the absence of a magnetic field the spectral function is the same for both spin orientations and the spin index α will be omitted. The fermionic excitations give the dominant contribution to the spectral function close to the spectral threshold. Thus, its most singular part can be expressed through the correlation function of the mobile impurity

operator as [19]

$$A_1(k,E) \propto \int \mathrm{d}t\mathrm{d}x e^{i(Et/\hbar - kx)} \left\langle d^\dagger(x,t)(0,0)\right\rangle \tag{8}$$

where the time evolution of the mobile-impurity hole operator is given by the model in Supplementary Eq. (2), $d(x,t) = \exp(-iH_{\mathrm{nTLL}}t/\hbar)\, d(x) \exp(iH_{\mathrm{nTLL}}t/\hbar)$, and the expectation value has to be taken also with respect to the whole model in Supplementary Eq. (2).

Integration over the bosonic field in Supplementary Eq. (8), in the same way as for the linear Tomonaga-Luttinger model, gives power-law singularities around the spectral edges,

$$A_1(k,E) \propto \frac{1}{|E \pm \varepsilon(k)|^{\alpha_\pm}}, \tag{9}$$

where the exponent depends on momentum [16, 20],

$$\alpha_\pm(k) = \frac{1 \mp 1}{2} - \frac{1}{2}\left(\frac{(2l+1)\sqrt{K_{\mathrm{c}}}}{\sqrt{2}} - C_+ - C_-\right)^2 - \frac{1}{2}\left(\frac{1}{\sqrt{2K_{\mathrm{c}}}} - C_+ - C_-\right). \tag{10}$$

Here $\pm$ refer to the particle (hole) sector on the energy-momentum plane and l is the integer number of translations of the principal region in the momentum variable from $-k_{\mathrm{F}}$ to k_{F} by $2k_{\mathrm{F}}$. Substitution of the parabolic dispersion for a repulsive spinful fermionic system obtained using Bethe ansatz method [21],

$$\varepsilon(k) = \mu + \frac{\hbar^2\left(k^2 - k_{\mathrm{F}}^2\right)}{2m_{1\mathrm{D}}K_s}, \tag{11}$$

where μ is the chemical potential, into Supplementary Eqs. (6) and (10) for $l = 0$ in the hole sector gives the explicit momentum dependence of the edge exponent quoted in Eq. (1) of the main text.

Supplementary References

[1] Tsyplyatyev, O. *et al.* Nature of the many-body excitations in a quantum wire: Theory and experiment. *Phys. Rev. B* **93**, 075147 (2016).

[2] Abrahams, E., Anderson, P. W., Licciardello, D. C. & Ramakrishnan, T. V. Scaling theory of localization: Absence of quantum diffusion in two dimensions. *Phys. Rev. Lett.* **42**, 673–676 (1979).

[3] Anderson, P. W. Absence of diffusion in certain random lattices. *Physical Review* **109**, 1492–1505 (1958).

[4] Altshuler, B. L. & Prigodin, V. N. Distribution of local density of states and NMR line shape in a one-dimensional conductor. *Journal of Experimental and Theoretical Physics* **68**, 198–209 (1989).

[5] Sanchez-Palencia, L. *et al.* Anderson localization of expanding Bose-Einstein condensates in random potentials. *Phys. Rev. Lett.* **98**, 210401–1–210401–4 (2007).

[6] Billy, J. *et al.* Direct observation of Anderson localization of matter waves in a controlled disorder. *Nature* **453**, 891–894 (2008).

[7] Schönhammer, K. & Meden, V. Nonuniversal spectral properties of the Luttinger model. *Phys. Rev. B* **47**, 16205–16215 (1992).

[8] Meden, V. Nonuniversality of the one-particle Greens function of a Luttinger liquid. *Phys. Rev. B* **60**, 4571–4575 (1999).

[9] Haldane, F. D. M. 'Luttinger liquid theory' of one-dimensional quantum fluids. i. properties of the Luttinger model and their extension to the general 1D interacting spinless fermi gas. *J. Phys. C: Solid State Phys.* **14**, 2585–2609 (1981).

[10] Samokhin, K. V. Lifetime of excitations in a clean Luttinger liquid. *J. Phys.: Condens. Matter* **10**, L533–L538 (1998).

[11] Imambekov, A. & Glazman, L. I. Universal theory of nonlinear Luttinger liquids. *Science* **323**, 228–231 (2009).

[12] Nozières, P. & De Dominicis, C. T. Singularities in the X-ray absorption and emission of metals. iii. one-body theory exact solution. *Physical Review* **178**, 1097–1107 (1969).

[13] Giamarchi, T. *Quantum physics in one dimension* (Clarendon press, Oxford, 2003).

[14] Pustilnik, M., Khodas, M., Kamenev, A. & Glazman, L. I. Dynamic response of one-dimensional interacting Fermions. *Phys. Rev. Lett.* **96**, 196405–1–196405–4 (2006).

[15] Khodas, M., Pustilnik, M., Kamenev, A. & Glazman, L. I. Spin-charge separation in one-dimensional fermion systems beyond the Luttinger liquid theory. *Physical Review B* **76**, 155402–1–155402–21 (2007).

[16] Schmidt, T. L., Imambekov, A. & Glazman, L. I. The fate of 1d spin-charge separation away from Fermi points. *Phys. Rev. Lett.* **104**, 116403-1–116403-4 (2010).

[17] Imambekov, A., Schmidt, T. L. & Glazman, L. I. One-dimensional quantum liquids: Beyond the Luttinger liquid paradigm. *Reviews of Modern Physics* **84**, 1253–1306 (2012).

[18] Abrikosov, A. A., Gorkov, L. P. & Dzyaloshinski, I. E. *Methods of quantum field theory in statistical physics* (Dover Publications, New York, 1975).

[19] Imambekov, A. & Glazman, L. I. Phenomenology of one-dimensional quantum liquids beyond the low-energy limit. *Phys. Rev. Lett.* **102**, 126405-1–126405-4 (2009).

[20] Schmidt, T. L., Imambekov, A. & Glazman, L. I. Fermi-Luttinger liquid: Spectral function of interacting one-dimensional fermions. *Physical Review B* **82**, 245104–1–245104–24 (2010).

[21] Tsyplyatyev, O. & Schofield, A. J. Spectral-edge mode in interacting one-dimensional systems. *Physical Review B* **90**, 014309-1–014309-9 (2014).
